# Crystalline Diuranium Phosphinidiide and μ‐Phosphido Complexes with Symmetric and Asymmetric UPU Cores

**DOI:** 10.1002/anie.201706002

**Published:** 2017-07-24

**Authors:** Thomas M. Rookes, Benedict M. Gardner, Gábor Balázs, Matthew Gregson, Floriana Tuna, Ashley J. Wooles, Manfred Scheer, Stephen T. Liddle

**Affiliations:** ^1^ School of Chemistry The University of Manchester Oxford Road Manchester M13 9PL UK; ^2^ Institute of Inorganic Chemistry University of Regensburg Universitätsstrasse 31 93053 Regensburg Germany

**Keywords:** density functional theory, metal–ligand multiple bonding, phosphido, phosphinidiide, uranium

## Abstract

Reaction of [U(Tren^TIPS^)(PH_2_)] (**1**, Tren^TIPS^=N(CH_2_CH_2_NSiPr^i^_3_)_3_) with C_6_H_5_CH_2_K and [U(Tren^TIPS^)(THF)][BPh_4_] (**2**) afforded a rare diuranium parent phosphinidiide complex [{U(Tren^TIPS^)}_2_(μ‐PH)] (**3**). Treatment of **3** with C_6_H_5_CH_2_K and two equivalents of benzo‐15‐crown‐5 ether (B15C5) gave the diuranium μ‐phosphido complex [{U(Tren^TIPS^)}_2_(μ‐P)][K(B15C5)_2_] (**4**). Alternatively, reaction of [U(Tren^TIPS^)(PH)][Na(12C4)_2_] (**5**, 12C4=12‐crown‐4 ether) with [U{N(CH_2_CH_2_NSiMe_2_Bu^t^)_2_CH_2_CH_2_NSi(Me)(CH_2_)(Bu^t^)}] (**6**) produced the diuranium μ‐phosphido complex [{U(Tren^TIPS^)}(μ‐P){U(Tren^DMBS^)}][Na(12C4)_2_] [**7**, Tren^DMBS^=N(CH_2_CH_2_NSiMe_2_Bu^t^)_3_]. Compounds **4** and **7** are unprecedented examples of uranium phosphido complexes outside of matrix isolation studies, and they rapidly decompose in solution underscoring the paucity of uranium phosphido complexes. Interestingly, **4** and **7** feature symmetric and asymmetric UPU cores, respectively, reflecting their differing steric profiles.

In recent years there has been burgeoning interest in the synthesis and chemistry of uranium–ligand multiple bonds,^1^ which stems from a desire to better understand the chemical bonding of uranium and to correlate this to observed physicochemical properties. However, most progress has been made regarding complexes where uranium engages in a formal multiple bond to C‐/N‐/O‐based donor ligands, and examples of second row‐centered, and beyond, donor ligands generally continue to be rare.^2^ Where uranium–phosphorus multiple bonding is concerned,^3^ only two structurally authenticated phosphinidene complexes have been reported,^4^ and investigations into uranium phosphido complexes are exceedingly rare and restricted to cryogenic matrix isolation and/or computational studies.^5^ Thus, there are no reports of uranium phosphido complexes on macroscopic scales under conditions that would permit further investigation; indeed, the phosphido linkage, whether terminal or μ‐bridging, remains a relatively rare structural motif even in transition‐metal chemistry.^6^

As part of our work on actinide–ligand multiple bonds,^7^ we reported dithorium phosphido and arsenido complexes that are supported by the very sterically demanding triamidoamine ligand N(CH_2_CH_2_NSiPr^i^_3_)_3_ (Tren^TIPS^).7a,7d For the ThPTh derivative this ligand combination produced a seemingly optimal balance of steric shielding of the ThPTh core versus inter‐Tren^TIPS^ steric repulsion. We therefore considered whether the analogous diuranium complex might be accessible; however, uranium has potentially deleterious and facile redox chemistry compared to the more redox‐robust thorium, and is smaller than thorium by 0.05–0.18 Å,^8^ so uranium with the same ligand set might well be too strained to form a stable UPU linkage and could very easily decompose. Herein, however, we report two different methods for the bulk‐scale preparation and subsequent characterization of diuranium μ‐phosphido complexes, utilizing Tren^TIPS^ and the related Tren^DMBS^ (Tren^DMBS^=N(CH_2_CH_2_NSiMe_2_Bu^t^)_3_) ligands, that are the first examples of uranium phosphido complexes outside of cryogenic spectroscopic experiments.5b,5c These complexes can be isolated and manipulated in the solid state, but we find that they are indeed highly sensitive and decompose rapidly in solution, which is in‐line with the prior absence of any synthetically accessible actinide phosphido complexes. Interestingly, depending on the steric profiles of the Tren ligands that support these phosphido complexes, symmetric and asymmetric UPU cores are observed in the solid state structures.

Our initial approach was to target a UP(H)U core via deprotonation/salt elimination and then effect deprotonation to give a phosphido complex. Accordingly, sequential treatment of the uranium(IV) phosphanide complex [U(Tren^TIPS^)(PH_2_)] (**1**)4a with benzyl potassium and then the separated ion pair [U(Tren^TIPS^)(THF)][BPh_4_] (**2**)4a afforded, after work‐up and recrystallization, dark red‐brown crystals of the diuranium(IV) parent phosphinidiide complex [{U(Tren^TIPS^)}_2_(μ‐PH)] (**3**) in 67 % isolated yield, Scheme 1.^9^ The synthesis of **3** requires **2** as elimination of KBPh_4_ is favorable owing to the outer sphere nature of the BPh_4_^−^ anion in **2** whereas any uranium‐coordinated halide is not displaced by the relatively soft P center.4a The ^1^H NMR spectrum of **3** spans the range −27 to +8 ppm and the ^29^Si NMR spectrum exhibits a single resonance at +11.6 ppm, which are both consistent with the uranium(IV) formulation of **3**.^10^ No ^31^P NMR resonance could be detected for **3**, most likely due to the phosphinidiide being bonded to two uranium(IV) ions. The ATR‐IR spectrum of **3** exhibits a weak, broad feature at approximately 2169 cm^−1^, consistent with the presence of the μ‐PH unit.^11^ SQUID magnetometry on powdered **3** gives magnetic moments of 4.3 and 0.8 μ_B_ at 298 and 2 K, respectively, with a steady fall in‐between these two extremes. These data are entirely consistent with the presence of two uranium(IV) ions in **3** and a low temperature magnetic moment that is tending to zero and dominated by temperature independent paramagnetism from the spin‐orbit coupled ground‐state multiplet of ^3^H_4_ uranium. A shoulder in the *χ* vs. *T* data is apparent at about 25 K which is most likely due to single‐ion crystal field effects rather than any magnetic exchange.^12^ Confirmation of the formulation of **3** was provided by the solid state crystal structure, Figure 1, which reveals U–P distances of 2.8187(12) and 2.8110(12) Å that, considering steric profiles, compares well to a U–P distance of 2.743(1) Å in [{U(C_5_Me_5_)_2_(OMe)}_2_(μ‐PH)]^11^ and the sum of the single‐bond covalent radii of uranium and phosphorus (2.81 Å).^8^

**Figure 1:**
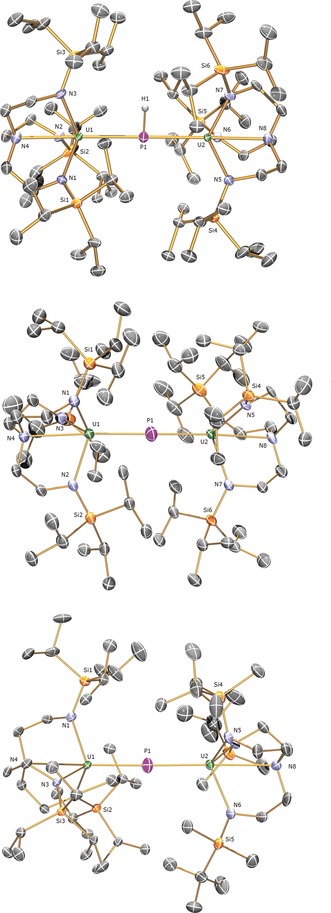
Molecular structures of **3** (top) and the anion components of **4** (middle), and **7** (bottom) at 150 K. Displacement ellipsoids set at 40 % probability and non‐phosphorus‐bound hydrogen atoms, minor disorder, and cation components are omitted for clarity. U green, P purple, N blue, Si yellow. Selected bond lengths [Å]: **3**, U1–P1 2.8187(12), U2–P1 2.8110(12), U1–N1 2.270(4), U1–N2 2.258(4), U1–N3 2.264(4), U1–N4 2.685(5), U2–N5 2.254(5), U2–N6 2.253(4), U2–N7 2.265(4), U2–N8 2.682(6); **4**, U1–P1 2.653(4), U2–P1 2.665(4), U1–N1 2.330(8), U1–N2 2.277(10), U1–N3 2.305(9), U1–N4 2.766(9), U2–N5 2.308(8), U2–N6 2.307(12), U2–N7 2.296(10), U2–N8 2.745(9); **7**, U1–P1 2.657(2), U2–P1 2.713(2), U1–N1 2.309(4), U1–N2 2.309(5), U1–N3 2.324(4), U1–N4 2.765(4), U2–N5 2.276(5), U2–N6 2.284(5), U2–N7 2.263(5), U2–N8 2.840(5).^20^

**Scheme 1:**
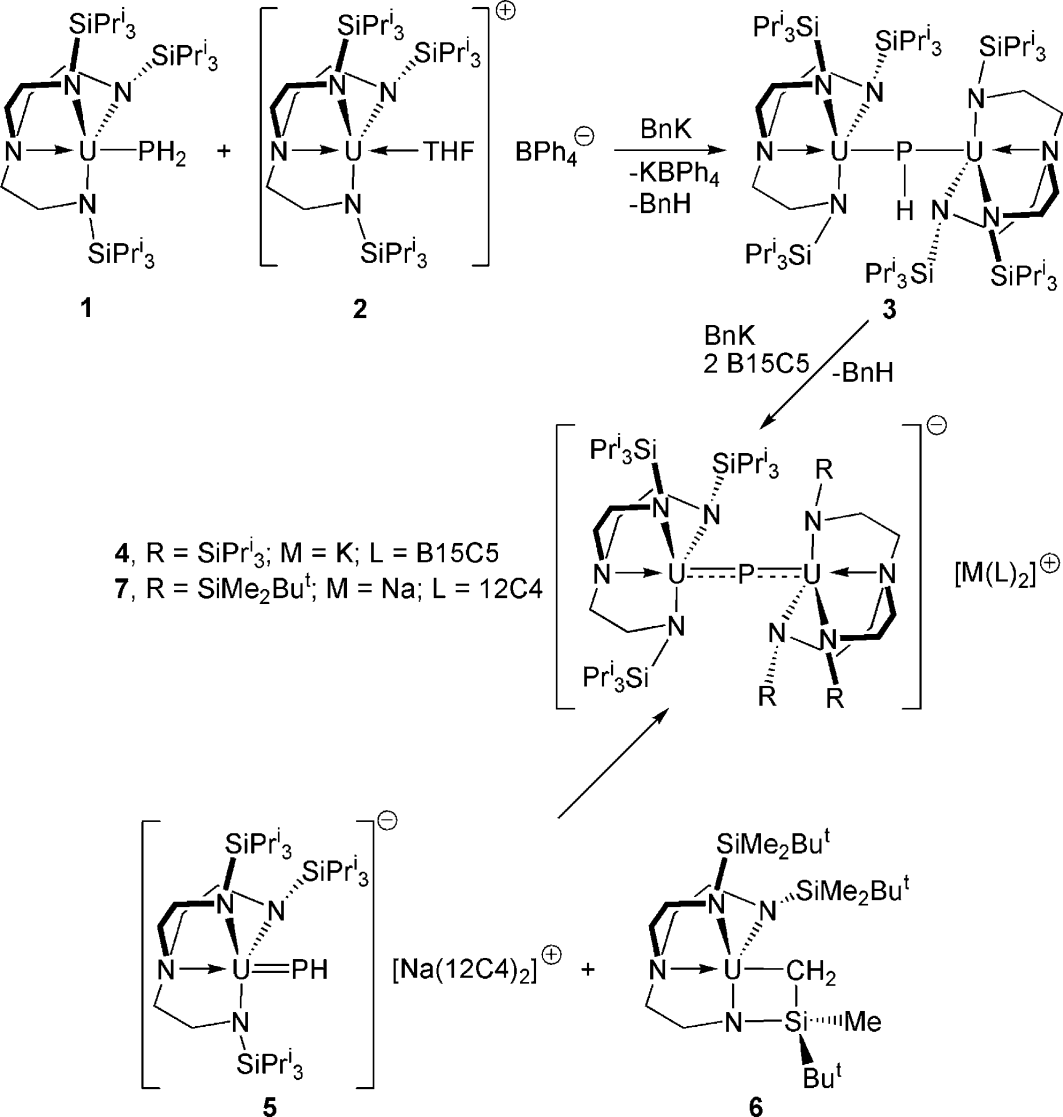
Synthesis of complex **3** from **1** and **2**, the conversion into **4**, and the formation of **7** from **5** and **6**. B15C5=benzo‐15‐crown‐5 ether, 12C4=12‐crown‐4 ether, Bn=benzyl.

With complex **3** secured, we attempted deprotonation of the phosphinidiide group. Treatment of **3** with one equivalent of benzyl potassium in the presence of two equivalents of benzo‐15‐crown‐5 ether (B15C5, to completely sequester the K ion) produced, after work‐up and recrystallization, a small crop (<5 % yield) of black crystals of the diuranium(IV) μ‐phosphido complex [{U(Tren^TIPS^)}_2_(μ‐P)][K(B15C5)_2_] (**4**), Scheme 1.^9^ The solid‐state crystal structure of **4**, Figure 1, confirms the separated ion pair formulation and reveals U–P distances of 2.653(4) and 2.665(4) Å, which represents a contraction of approximately 0.15 Å from **3**. The U–P distances in **4** can be considered to be short when considering the bridging nature of the phosphido; for example, although the sum of the covalent uranium and phosphorus double bond radii is 2.36 Å,^8^ the U=P distances in the terminal uranium(IV) phosphinidene complexes [U(Tren^TIPS^)(PH)][K(B15C5)_2_]4a and [U(C_5_Me_5_)_2_(P‐2,4,6‐Bu^t^_3_C_6_H_2_)(OPMe_3_)]4b are 2.613(2) and 2.562(3) Å, respectively. Furthermore, the Th–P distances in [{Th(Tren^TIPS^)}_2_(μ‐P)][Na(12C4)_2_] (12C4=12‐crown‐4 ether)7d are significantly longer [2.735(2)/2.740(2) Å] than the U–P distances in **4**, even when factoring in the covalent radii differences between thorium and uranium.^8^ The U–N_amide_ distances are around 0.1 Å longer than is typical for uranium(IV) Tren complexes,^13^ reflecting the anionic formulation of the phosphido moiety. We note that the U–N_amine_ distances are long, which infers a *trans*‐influence from the phosphido ligand,7d but this cannot be stated with confidence due to U−N bond lengthening from the anionic formulation.

Complex **4** decomposes in solution, which, together with the low yield, precluded further characterization beyond the X‐ray crystal structure and elemental analyses. The reaction that produces **4** is highly capricious, and despite exhaustive attempts the reaction conditions could not be improved; sometimes deprotonation of **3** fails, or complete decomposition occurs to unidentified products. Use of different organo‐alkali‐metal reagents, the presence or absence of different crown ethers, or increasing the molar quantity of benzyl potassium results in intractable reaction mixtures and/or production of the known uranium(IV) cyclometallate complex [U{N(CH_2_CH_2_NSiPr^i^_3_)_2_(CH_2_CH_2_NSiPr^i^_2_C(H)(Me)(CH_2_)}],^14^ where the fate of the phosphorus‐containing products could not be determined.

The above mentioned observations likely reflect the inherently polarized, weak, and labile nature of these U–P linkages, as reflected by the paucity of any other macroscopic molecular uranium phosphido complexes, and also likely steric overloading from close proximity of two Tren^TIPS^ ligands. In order to reduce this steric strain and perhaps obtain a more tractable phosphido complex, we adopted a different strategy to introduce a sterically less demanding Tren ligand.

Reaction of the new terminal uranium(IV)‐phosphinidene complex [U(Tren^TIPS^)(PH)][Na(12C4)_2_] (**5**),^9^ which is only the third example of a uranium phosphinidene, with the uranium(IV) cyclometallate complex [U{N(CH_2_CH_2_NSiMe_2_Bu^t^)_2_CH_2_CH_2_NSi(Me)(CH_2_)(Bu^t^)}] (**6**)^15^ proceeds by protonolysis to give the diuranium μ‐phosphido complex [{U(Tren^TIPS^)}(μ‐P){U(Tren^DMBS^)}][Na(12C4)_2_] (**7**), isolated as dark brown crystals in 29 % yield, Scheme 1.^9^ The crystalline yield is low due to the oily nature of **7**, and the decomposition that occurs once it is formed (see below). The solid‐state crystal structure of **7**, Figure 1, is in gross terms very similar to that of **4**, noting the change of Tren ligand and cation component. However, the U–P distances of 2.657(2) and 2.713(2) Å are notable in that the shorter is consistent with the U–P distances in **4**, but the longer is significantly longer and mid‐way to the U–P distances in **3**. Interestingly, the shorter U–P distance is found for the Tren^TIPS^‐bound uranium with the longer U–P distance associated with the sterically less demanding Tren^DMBS^ portion, and the U−N bonds are longer in the Tren^TIPS^U portion of the molecule compared to those in the Tren^DMBS^U fragment, perhaps reflecting the asymmetry of the phosphido bonding.

The presence of uranium(IV) ions in **7** was confirmed by SQUID magnetometry on a powdered sample of **7**; the magnetic moments of 4.3 and 1.1 μ_B_ at 298 and 2 K, respectively, are consistent with the presence of uranium(IV) ions. However the magnetic moment of **7** at 2 K is higher than the corresponding data for **3**, which may represent the relative crystal‐field effects on uranium(IV) from HP^2−^ versus P^3−^; the P^3−^ would be expected to present a greater point charge and splitting of the paramagnetic excited states manifold, so a low‐lying group are still populated to some extent at low temperature with a higher‐lying group at high temperature that are more difficult to populate. This notion is consistent with a slightly flatter magnetic trace at high temperature for **7** compared to **3** and has been noted in other uranium(IV) complexes with strong point‐charge ligands.2h, 3b, 7f,7h, 16 Interestingly, counter to expectations the shoulder at about 25 K for the magnetic data of **3** is much less pronounced for **7** which is consistent with our suggestion that this feature is due to single ion crystal field effects and not magnetic exchange,^12^ though magnetic exchange cannot be completely ruled out.

Complex **7** is moderately more stable than **4**, but although, once isolated, solid state characterization methods were feasible we find that redissolving **7** results in rapid decomposition so NMR and optical spectroscopic data were unobtainable. Interestingly, we find that the majority decomposition products of **7** are the uranium(IV)‐cyclometallate complex [U{N(CH_2_CH_2_NSiPr^i^_3_)_2_(CH_2_CH_2_NSiPr^i^_2_C(H)(Me)(CH_2_)}],^14^ and what we deduce to be [U(Tren^DMBS^)(PH)][Na(12C4)_2_], though the latter is not sufficiently sterically protected so decomposes to unidentified products. Nevertheless, the more clear‐cut nature of the decomposition of **7** compared to **4** is instructive because it suggests that even with reduced ligand steric demands the UPU unit is inherently unstable. Interestingly, the decomposition reaction of **7** produces a cyclometallate with a less‐strained 5‐membered metallocyclic ring compared to the more‐strained 4‐membered metallocycle in **6**. This aspect is also consistent with the observation that mixing the five‐membered‐ring cyclometallate [U{N(CH_2_CH_2_NSiPr^i^_3_)_2_(CH_2_CH_2_NSiPr^i^_2_C(H)(Me)(CH_2_)}]^14^ and known [U(Tren^TIPS^)(PH)][K(B15C5)_2_]4a gives no reaction. Thus, the importance of metallocyclic ring‐strain as a key factor in driving the protonolysis reaction to generate **7** emerges. This point is underscored when considering that on the basis of the solid‐state structure the phosphido appears to be more associated with the Tren^TIPS^U fragment rather than the Tren^DMBS^U group, but it is the Tren^TIPS^U fragment that is, in essence, the leaving group during decomposition.

To gain a greater understanding of the bonding in the UPU units of **4** and **7**, we carried out DFT calculations on the full anion components of these compounds, **4** ^−^ and **7** ^−^, respectively. Considerable difficulty was encountered obtaining SCF‐converged structures, which suggests that **4** ^−^ and **7** ^−^ have multi‐reference ground states. However, satisfactorily converged models that provide a qualitative description of the electronic structure of these compounds could be obtained.

Both **4** ^−^ and **7** ^−^ exhibit four unpaired electrons of essentially exclusive 5f character in their α‐spin manifolds as HOMO to HOMO−3, which is consistent with the presence of two 5f^2^ uranium(IV) ions. HOMO−4 to HOMO−6 in each case represent the principal bonding components in the UPU units, see Figure 2 and the Supporting Information,^9^ confirming the presence of polarized uranium–phosphido triple bonding interactions. The uranium spin densities of −2.31/−2.33 and −2.17/−2.22, for **4** ^−^ and **7** ^−^ respectively, show donation of electron density from the ligands to uranium and support the uranium(IV) formulations. The uranium charges are high for Tren uranium(IV) complexes,^17^ at +3.79/+3.86 for **4** ^−^ and +3.52/+3.87 for **7** ^−^ and the phosphido charges are −2.19 and −2.35, respectively. Interestingly, the uranium ion in **7** ^−^ which has the closest association with the phosphido, that is, Tren^TIPS^U, has the highest charge and lowest spin density, and recall that the U–N distances are longer for that unit than the Tren^DMBS^U unit; this suggests that the N atoms are better as a unit at charge donation to uranium than the phosphido.^18^

**Figure 2:**
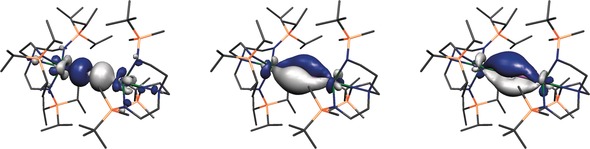
Kohn–Sham frontier molecular orbitals of that represent the principal bonding components of the UPU unit in the anion component of **7**, **7** ^−^: Left, HOMO−6 (403a, −1.332 eV); Middle, HOMO−5 (404a, −0.997 eV); Right, HOMO−4 (405a, −0.983 eV). Hydrogen atoms are omitted for clarity.

The UP Mayer bond orders reflect multiple, but polarized bond interactions. Specifically, in **4** ^−^ they are 1.41/1.43 whereas for **7** ^−^ they are 1.44/1.66 reflecting the asymmetric UP distances and bonding in the UPU core in **7** ^−^; notably, these UP bond orders are in‐line with the situation in the Lewis bonding scheme for these units, that is, U=P=U. These Mayer bond orders should be viewed in the context that the UN_amide_ and UN_amine_ bond orders are 0.71 and 0.18, respectively, and they are surprisingly invariant across **4** ^−^ and **7** ^−^.

The bond topological data are remarkably invariant, showing polar, quite ionic UP bonds with *ρ* values of 0.06 (typically *ρ*>0.1 for covalent bonds) and bond ellipticities that are zero or close to zero^9^ reflecting the formal triple bond interactions that constitute cylindrical distributions of electron density with respect to the inter‐nuclear axes.^19^ Polar UP bonding is also suggested by NBO analyses, which finds UP σ‐bonds with 16 % U and 84 % P character (U: 1:1:69:29 % 7s:7p:5f:6d; P: 100 % 3p) and UP π‐bonds with 26 % U and 74 % P character (U: 0:1:54:45 7s:7p:5f:6d; P: 100 % 3p).

The data above unequivocally suggest that the UPU interactions in **4** ^−^ and **7** ^−^ are polarized and weak, which is consistent with the observed instability of **4** and **7**. Interestingly, the UP bonds for **4** ^−^ and **7** ^−^ have higher Mayer bond orders, exhibit more metal component, and utilize more 5f character (relative to 6d) than the ThP bonds in [{Th(Tren^TIPS^)}_2_(μ‐P)][Na(12C4)_2_],7d consistent with the general view that uranium engages in more covalent bonding, and with greater 5f character, than thorium, but we note that the bond topological data are essentially invariant for uranium and thorium. This suggests that the instability of **4** and **7** is most likely of kinetic origin.

To conclude, we have reported two structurally authenticated examples of uranium phosphido complexes. These linkages are unprecedented outside of cryogenic matrix isolation conditions, remain rare even in the d‐block, and indeed uranium–phosphorus multiple bonding remains exceedingly rare overall. These complexes have been prepared on macroscopic scales by two different methodologies that could greatly expand uranium–phosphido chemistry: 1) construction of a UP(H)U unit by salt elimination and subsequent deprotonation; or 2) protonation of a cyclometallate by a parent phosphinidene. Although both complexes can be prepared and isolated they exhibit intrinsic instability that is consistently reflected in quantum chemical calculations. Low‐temperature magnetism studies also suggest differences in the relative crystal‐field effects on uranium(IV) from HP^2−^ versus P^3−^. Most intriguingly, the UP bond lengths can be perturbed by co‐ligand steric demands, which suggests that with suitably chosen co‐ligands perhaps a UPU linkage, or perhaps a UPM unit that might be prepared by method (2), could be polarized to the point of rupture in order to produce a terminal uranium phosphido complex under ambient conditions. Efforts in that regard are on‐going.

## Conflict of interest

The authors declare no conflict of interest.

## Supporting information

As a service to our authors and readers, this journal provides supporting information supplied by the authors. Such materials are peer reviewed and may be re‐organized for online delivery, but are not copy‐edited or typeset. Technical support issues arising from supporting information (other than missing files) should be addressed to the authors.

Supplementary
