# Supplementary material for: Crystalline Diuranium Phosphinidiide and μ‐Phosphido Complexes with Symmetric and Asymmetric UPU Cores
Source: Angew Chem Int Ed Engl. 2017 Jul 24;56(35):10495–500. doi: 10.1002/anie.201706002 (PMC5577518; doi:10.1002/anie.201706002)
Supplement: Supplementary file 1 — Supplementary [file ANIE-56-10495-s001.pdf]

Angewandte Chemie
Eine Zeitschrift der Gesellschaft Deutscher Chemiker

Supporting Information

Crystalline Diuranium Phosphinidiide and μ-Phosphido Complexes with Symmetric and Asymmetric UPU Cores

Thomas M. Rookes, Benedict M. Gardner, Gábor Balázs, Matthew Gregson, Floriana Tuna, Ashley J. Wooles, Manfred Scheer, and Stephen T. Liddle**

anie_201706002_sm_miscellaneous_information.pdf

Experimental

General

All manipulations were carried out using Schlenk techniques, or an MBraun UniLab glovebox, under an atmosphere of dry nitrogen. Solvents were dried by passage through activated alumina towers and degassed before use or were distilled from calcium hydride. All solvents were stored over potassium mirrors, except for ethers that were stored over activated 4 Å sieves. Deuterated solvent was distilled from potassium, degassed by three freeze-pump-thaw cycles and stored under nitrogen. $[U\{N(CH_2CH_2NSiPr^i_3)_2(CH_2CH_2NSiPr^i_2C[H]MeCH_2)\}]$, $[U\{N(CH_2CH_2NSi^tBuMe_2)_2(CH_2CH_2NSi^tBuMeCH_2\}]$, $[U(Tren^{TIPS})(THF)][BPh_4]$, $[U(Tren^{DMBS})(PH_2)]$, $NaPH_2$, and $[KCH_2C_6H_5]$ were prepared according to literature procedures.[1-6] Benzo-15-crown-5 ether (B15C5) was dissolved in ether, dried over 4 Å sieves, decanted and solvent removed. 12-crown-4 ether was dried over 4 Å sieves. ^{1}H, ^{13}C, ^{29}Si, and ^{31}P NMR spectra were recorded on a Bruker 400 spectrometer operating at 400.2, 100.6, 79.5, and 162.0 MHz respectively; chemical shifts are quoted in ppm and are relative to TMS (^{1}H, ^{13}C, ^{29}Si) and 85% H_3PO_4 (^{31}P). FTIR spectra were recorded on a Bruker Tensor 27 spectrometer. Variable-temperature magnetic moment data were recorded in an applied dc field of 0.1 T on a Quantum Design MPMS XL7 SQUID magnetometer using doubly recrystallised powdered samples. Care was taken to ensure complete thermalisation of the sample before each data point was measured and samples were immobilised in an eicosane matrix to prevent sample reorientation during measurements. Diamagnetic corrections were applied using tabulated Pascal constants and measurements were corrected for the effect of the blank sample holders (flame sealed Wilmad NMR tube and straw) and eicosane matrix. CHN microanalyses were carried out by Mr M Jennings at the University of Manchester and by Tong Liu at the University of Nottingham. Persistently low C% values plagued some compounds due to incomplete combustion and SiC formation.[7]

Preparation of [{U(Tren^{TIPS})}$_2$(μ-PH)] (3)

Toluene (40 ml) was added to a cold (–78 °C) mixture of **1** (2.61 g, 2.95 mmol) and benzylpotassium (0.39 g, 2.95 mmol). The dark mixture was allowed to warm to room temperature while stirring over 2 hours. Volatiles were removed *in vacuo* to afford a sticky dark brown solid. Complex **2** (3.66 g, 2.95 mmol) was added, the mixture was cooled to –78 °C and THF was added. The suspension was allowed to warm to ambient temperature and stirred for 16 hours. The resulting thick brown slurry was reduced to dryness *in vacuo*, extracted with 2 × 40 ml hot (80 °C) toluene and filtered through a frit to afford a dark green-brown solution. Volatiles were removed under reduced pressure and the dark red-brown solid was washed thoroughly with pentane (4 × 20 ml) and dried *in vacuo* for 1 hour. Single crystals of **3** suitable for single crystal X-ray diffraction were grown from a saturated solution in THF at 5 °C overnight. Yield: 3.42 g (67%). Anal. calc'd for $C_{66}H_{151}N_8PSi_6U_2$·(3.1THF): C 48.14%; H 9.06%; N 5.73%. Found: C 48.39%; H 8.81%; N 5.99 %. ^{1}H NMR (THF-d_8, 298 K): δ –27.04 (s, 6H, CH_2), –15.45 (s, 6H, CH_2), –7.10 (s, 6H, CH_2), –1.50 (s, 6H, CH_2), 3.52 (s, 18H, C*H*(CH_3)$_2$), 4.79 (s, br, FWHM = 96 Hz, 54H, CH(*CH_3*)$_2$), 7.93 (s, br, FWHM = 100 Hz, 54H, CH(*CH_3*)$_3$). ^{29}Si{^{1}H} NMR (THF-d_8, 298 K): δ 11.63. ^{31}P NMR signal not observed. ATR-IR (cm^{-1}): *v* 2938 (m), 2862 (m), 2840 (m), 2169 (w,br), 1452 (w), 1379 (w), 1361 (w), 1335 (w), 1271 (w), 1241 (w), 1142 (w), 1037 (m), 1014 (m), 990 (w), 929 (s), 912 (m), 878 (s), 798 (m), 721 (s), 671 (s), 649 (s), 632 (s), 567 (m), 552 (m), 512 (w), 461 (w).

Preparation of [{U(Tren^{TIPS})}$_2$(μ-P)][K(B15C5)$_2$] (4)

Toluene (40 ml) was added to a cold (–78 °C) mixture of **3** (3.42 g, 1.97 mmol) and benzylpotassium (0.26 g, 1.97 mmol). The dark suspension was allowed to warm to ambient temperature while stirring over 1 hour and stirred for a further 16 hours. The resulting dark brown suspension was cooled to –78 °C and benzo-15-crown-5 ether (1.06 g, 3.94 mmol) in

toluene (*ca.* 10 ml) was added and allowed to warm to ambient temperature while stirring over 1 hour before being stirred for a further 16 hours. The resulting brown-black mixture was gently warmed to 50 °C and filtered whilst still warm. The volume of solvent was reduced *in vacuo* to 8 ml and stored at –30 °C overnight to afford a small crop of black crystals of **4** suitable for single crystal X-ray diffraction (<5% yield). Anal. calc'd for $C_{94}H_{190}KN_8O_{10}PSi_6U_2$: C 48.93%; H 8.30%; N 4.86%. Found: C 48.59%; H 8.15%; N 4.63%. Complex **4** decomposes rapidly in solution to unidentified products, which precluded the acquisition of NMR and UV/Vis/NIR spectroscopic data. The low yield precluded the acquisition of IR data.

Preparation of [U(Tren^{TIPS})(PH)][Na(12C4)$_2$] (5)

A THF (40 ml) solution of 12-crown-4 ether (1.76 g, 10.0 mmol) was added to a cold (−78 °C) mixture of $[U\{N(CH_2CH_2NSiPr^i_3)_2(CH_2CH_2NSiPr^i_2C(H)(Me)(CH_2)\}]$ (4.25 g, 5.0 mmol) and $NaPH_2$ (0.28 g, 5.0 mmol). The resulting dark brown solution was stirred at −78 °C for 15 min and allowed to warm to room temperature after which volatiles were removed *in vacuo* to afford a sticky black solid. The product was thoroughly washed with pentane (4 × 20 ml) and subsequently extracted with toluene (2 × 20 ml) and filtered. Solvents were removed *in vacuo* the resulting black solid was further washed with hexane (2 × 10 ml) and dried *in vacuo* for 1 hr yielding a black crystalline solid. Single crystals of **5** suitable for single crystal X-ray diffraction were grown from a saturated toluene solution stored at –30 °C for 16 hrs. Yield 4.62 g (73 %). Anal. calc'd for $C_{56}H_{116}N_4NaO_8PSi_3U_2$: C 46.80%; H 8.66%; N 4.45%. Found: C 45.51%; H 8.53%; N 4.64% ^{1}H NMR (C_6D_6, 298 K): δ −8.81 (b, 54H, CH(CH_3)$_2$), −7.44 (b, 9H, C*H*(CH_3)$_2$), −0.81 (s, 6H, C*H*$_2$), 4.97 (s, 16H, CH_2CH_2O), 7.24 (s, 16H, CH_2CH_2O), 10.17 (s, 6H, C*H*$_2$). ^{31}P and ^{29}Si NMR signals not observed. ATR-IR (cm^{-1}): *ν* 2858 (s), 1461 (s), 1445 (s), 1401 (w), 1379 (w), 1364 (s), 1335 (s), 1304 (w), 1289 (s),

1273 (w), 1246 (s), 1135 (s), 1094 (s), 1048 (s), 1021 (s), 990 (w), 930 (s), 916 (s), 880 (s), 849 (s), 796 (s), 730 (s), 668 (s), 625 (s), 554 (s), 511 (s), 444 (m). The region 2320-2050 cm^{-1} contains multiple weak absorptions; we believe that in this instance the PH stretch is coupled to other vibrations in the compound, a common phenomenon for heavy-p-block complexes, so a precise stretching frequency cannot be assigned.

Preparation of [U(Tren^{TIPS})(Tren^{DMBS})(μ-P)][Na(12C4)$_2$] (7)

THF (40 ml) was added to a cold (–78 °C) mixture of **5** (0.7 g, 0.55 mmol) and **6** (0.40 g, 0.55 mmol). The dark brown solution was allowed to warm to room temperature and stirred overnight. Solvents were removed *in vaccuo* and the resulting dark brown solid was extracted with toluene and filtered. The solvents were removed *in vaccuo* and the black solid was washed with pentane (2 x 10 ml). Single crystals of **7** suitable for single crystal X-ray diffraction were obtained from a concentrated toluene solution stored at –30 °C for 48 hrs. Yield 0.32 g (29 %). Anal. calc'd for $C_{73}H_{164}N_8NaO_8PSi_6U_2$: C 44.27; H 8.35; N 5.66 %. Found C 43.80, H 8.50, N 5.74 %. Complex **7** decomposes in solution to $[U\{N(CH_2CH_2NSiPr^i_3)_2(CH_2CH_2NSiPr^i_2C(H)(Me)(CH_2)\}]$ and unidentified products which precluded the acquisition of NMR and UV/Vis/NIR spectroscopic data. ATR-IR (cm^{-1}): *ν* 2921 (s), 2855 (s), 1972 (w), 1462 (m), 1401 (m), 1383 (w), 1364 (w), 1334 (m), 1303 (w), 1287 (m), 1244 (s), 1135 (s), 1960 (s), 1508 (m), 1022 (s), 933 (s), 917 (s), 881 (s), 850 (s), 821 (2), 792 (m), 770 (m), 733 (s), 654 (s), 515 (m), 454 (w), 437 (w), 405 (w).

Magnetometric Data

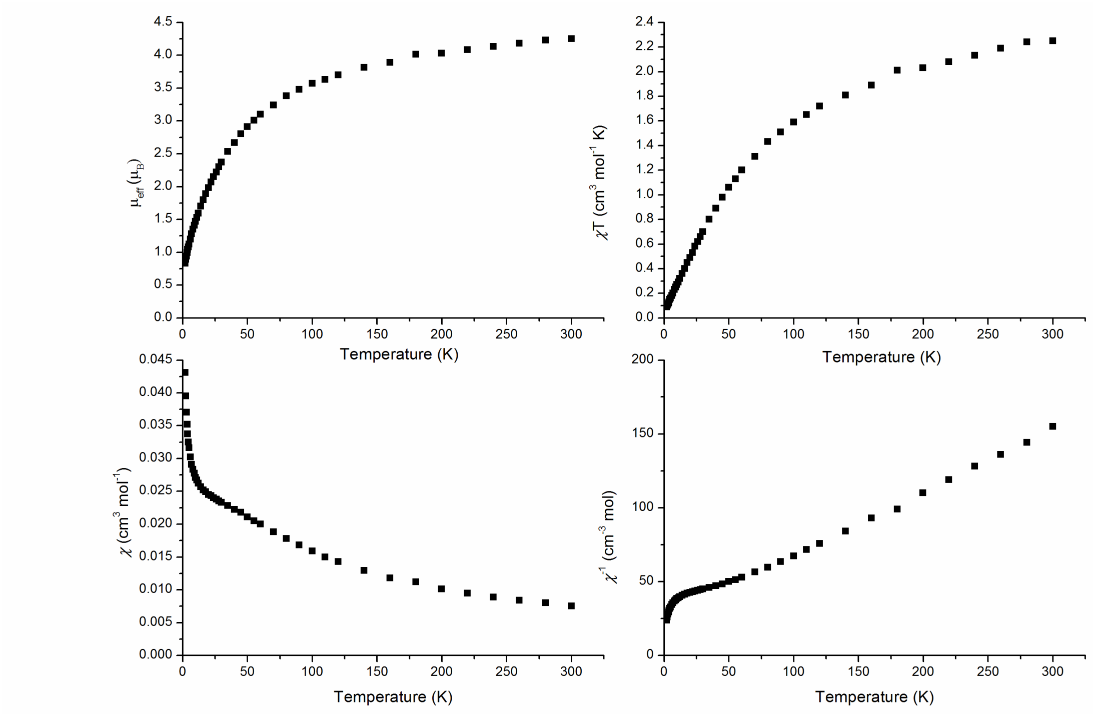

Figure S1. μ_{eff}, χT, χ, and $1/\chi$ vs T data for **3**.

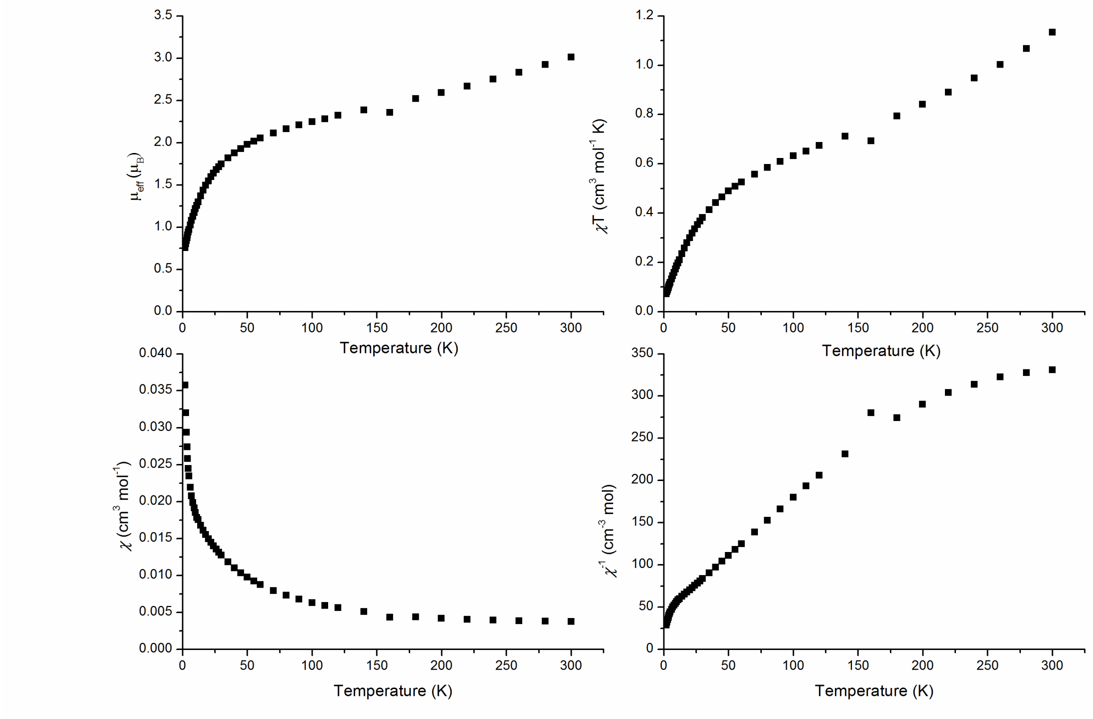

Figure S2. μ_{eff}, χT, χ, and $1/\chi$ vs T data for **5**.

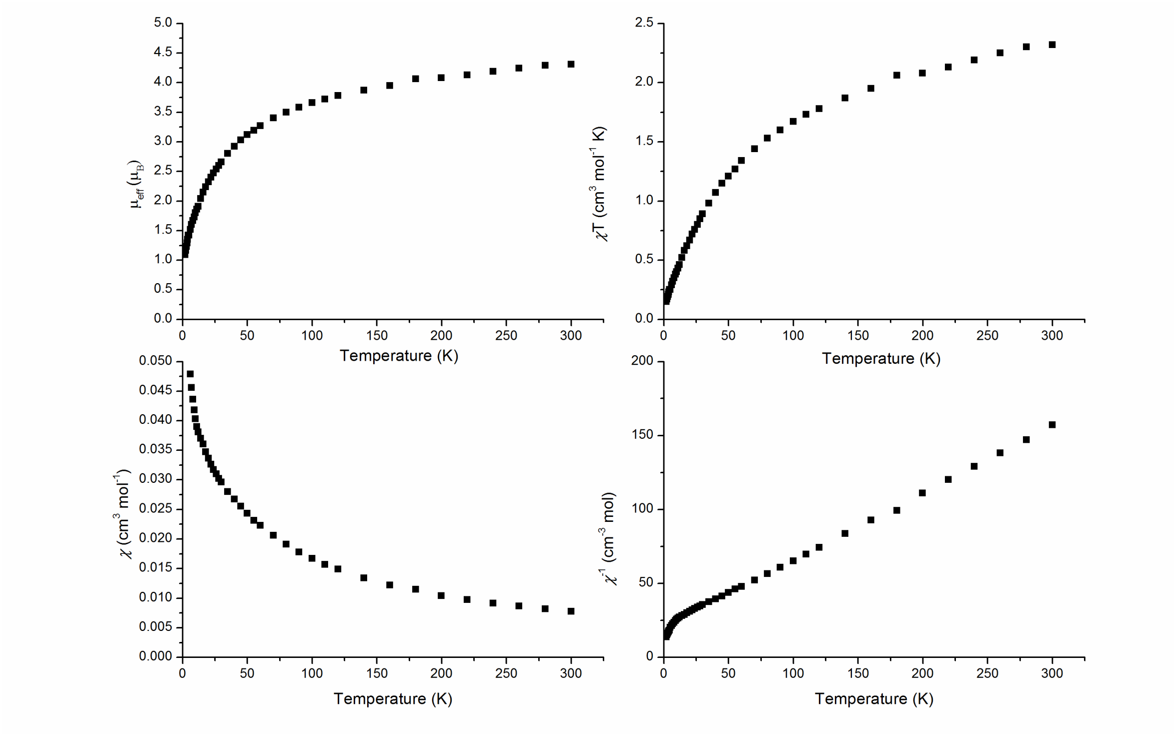

Figure S3. μ_{eff}, χT, χ, and $1/\chi$ vs T data for **7**.

X-ray Crystallography (CCDC numbers 1554770-1554773)

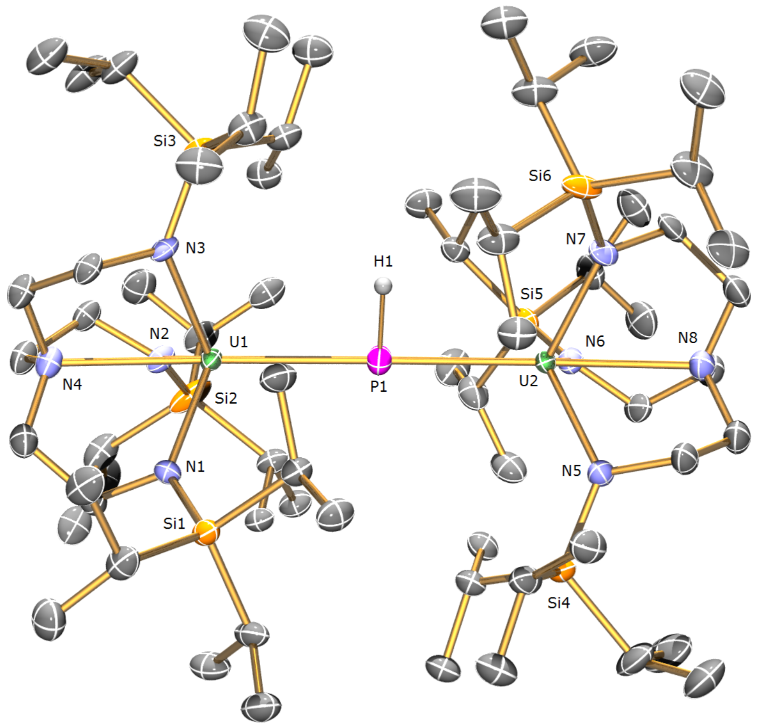

Figure S4. Molecular structure of **3** at 150 K. Displacement ellipsoids set at 40% and non-phosphorus-bound hydrogen atoms and minor disorder components omitted for clarity.

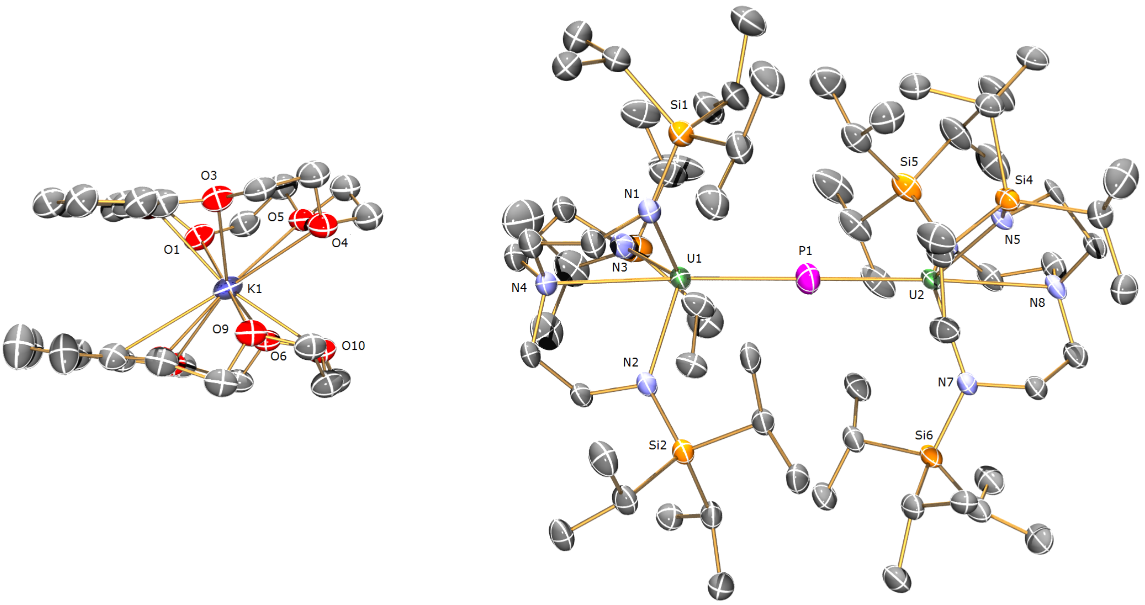

Figure S5. Molecular structure of **4** at 150 K. Displacement ellipsoids set at 40% and hydrogen atoms and minor disorder components omitted for clarity.

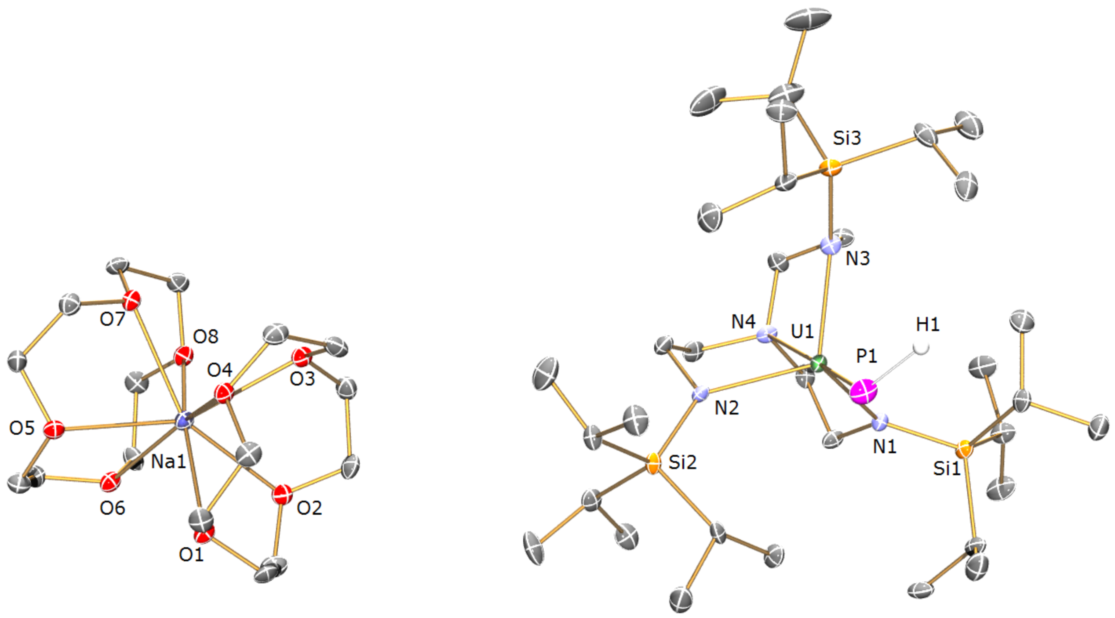

Figure S6. Molecular structure of **5** at 150 K. Displacement ellipsoids set at 40% and non-phosphorus-bound hydrogen atoms and minor disorder components omitted for clarity.

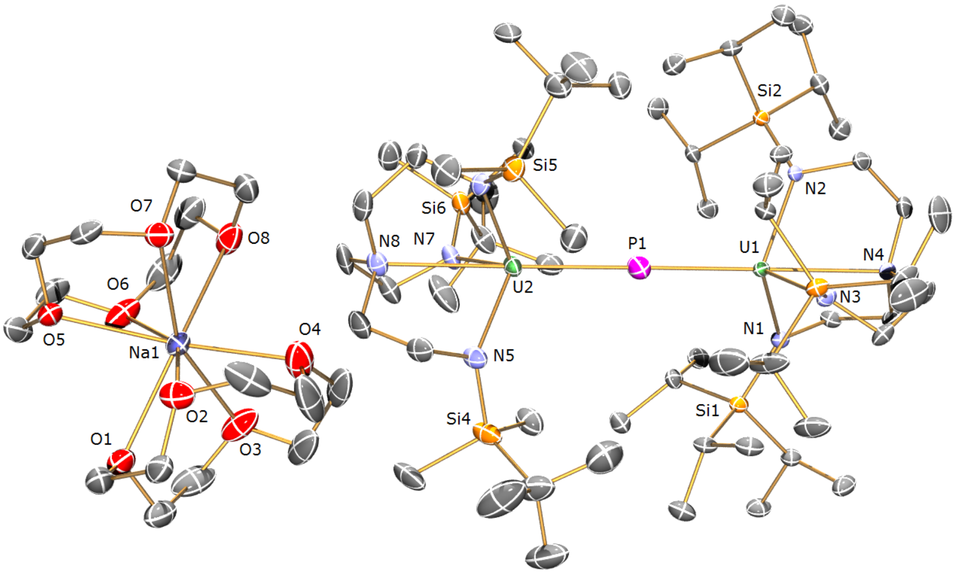

Figure S7. Molecular structure of **7** at 150 K. Displacement ellipsoids set at 40% and hydrogen atoms and minor disorder components omitted for clarity.

Computational Studies

General

Unrestricted geometry optimisations were performed for the full models of $\mathbf{4}^{-}$ and $\mathbf{7}^{-}$ using coordinates derived from the X-ray crystal structures. Considerable issues were encountered in obtaining converged SCFs due to the presence of four unpaired electrons in nearly degenerate essentially non-bonding orbitals in each complex anion. For $\mathbf{4}^{-}$ this required geometry optimisations to be run using the 'smearq' command, to initially smear the 5f-electron density around the 5f manifold, which was tightened up after each geometry optimisation iteration until eventually a converged, Aufbau-configuration geometry was finally run through a single point energy calculation with the α- and β-spin manifold occupations fixed. For $\mathbf{7}^{-}$, intractable convergence issues were encountered despite numerous attempts to follow the strategy that worked for $\mathbf{4}^{-}$. However, a single point energy calculation on the crystallographic coordinates with fixed occupations in the α- and β-spin manifolds

produced a satisfactorily converged SCF. Otherwise, no other constraints were imposed on the calculations. The calculations were performed using the Amsterdam Density Functional (ADF) suite version 2012.01.[8,9] The DFT geometry optimisations employed Slater type orbital (STO) triple-ζ-plus polarisation all-electron basis sets (from the ZORA/TZP database of the ADF suite). Scalar relativistic approaches were used within the ZORA Hamiltonian for the inclusion of relativistic effects and the local density approximation (LDA) with the correlation potential due to Vosko et al[10] was used in all of the calculations. Gradient corrections were performed using the functionals of Becke[11] and Perdew.[12] MOLEKEL[13] was used to prepare the three-dimensional plot of the electron density. Natural Bond Order (NBO) analyses were carried out with NBO 5.0.[14] The Atoms in Molecules analysis[15,16] was carried out with Xaim-1.0.[17]

Selected Data for 4^- and 7^-

4^-: Charge on U = +3.79/+3.86; Charge on P = −2.19; Spin Density on U = −2.31/−2.33; UP Mayer Bond Orders = 1.41/1.43; UN_{amide} Mayer Bond Orders (av.) = 0.71; UN_{amine} Mayer Bond Orders = 0.18; UP ρ = 0.06/0.06; UP $\nabla^2\rho$ = 0.10/0.10; UP ε = 0.10/0.12.

7^-: Charge on U (av.) = +3.52/+3.87; Charge on P = −2.35; Spin Density on U (av.) = −2.17/−2.22; UP Mayer Bond Orders = 1.44/1.66; UN_{amide} Mayer Bond Orders (av.) = 0.71; UN_{amine} Mayer Bond Orders = 0.18; UP ρ = 0.06/0.06; UP $\nabla^2\rho$ = 0.10/0.10; UP ε = 0.01/0.02.

Selected Kohn Sham Molecular Orbitals for 4⁻

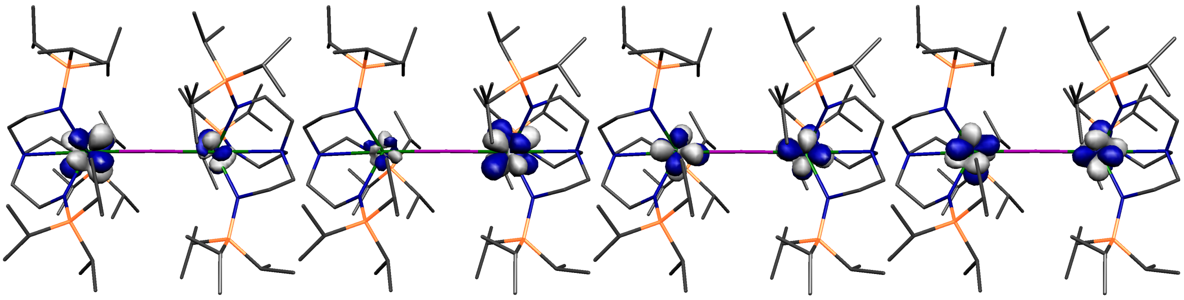

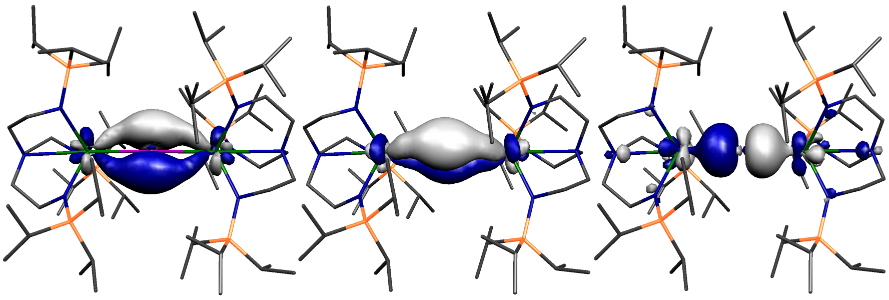

Figure S8. Top, left to right: HOMO (445a, −0.295 eV), HOMO−1 (444a, −0.301 eV), HOMO−2 (443a, −0.329 eV), HOMO−3 (442a, −0.341 eV). Bottom, left to right: HOMO−4 (441a, −1.291), HOMO−5 (440a, −1.296), HOMO−6 (439a, −1.828 eV). Hydrogen atoms are omitted for clarity.

Selected Kohn Sham Molecular Orbitals for 7

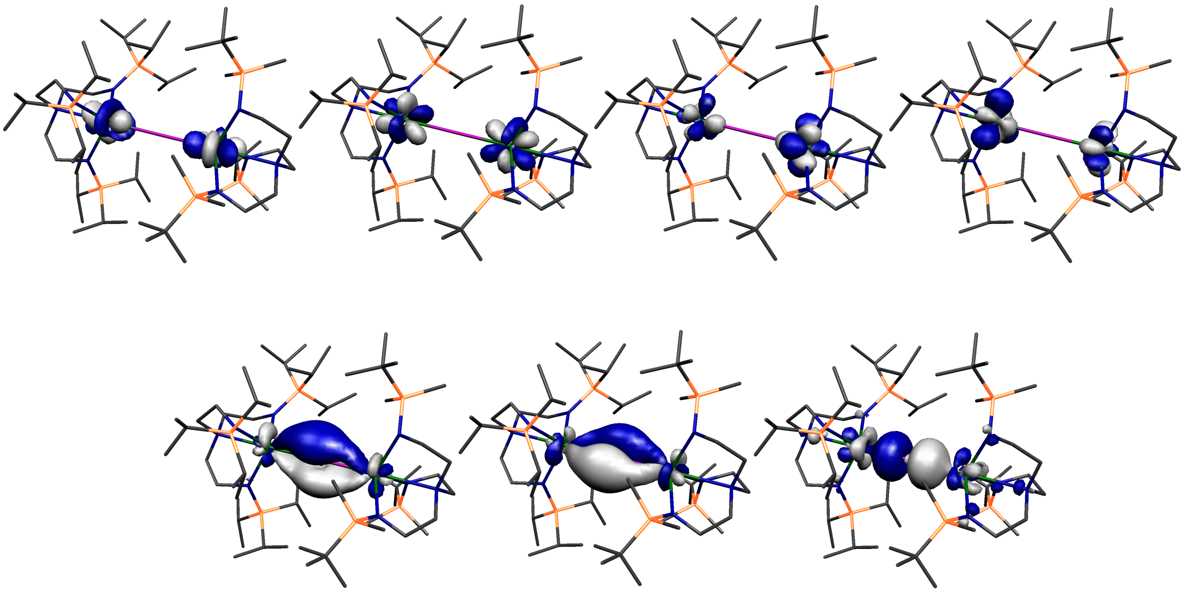

Figure S9. Top, left to right: HOMO (409a, 0.147 eV), HOMO−1 (408a, 0.112 eV), HOMO−2 (407a, 0.091 eV), HOMO−3 (406a, 0.057 eV). Bottom, left to right: HOMO−4 (405a, −0.983), HOMO−5 (404a, −0.997), HOMO−6 (403a, −1.332 eV). Hydrogen atoms are omitted for clarity.

Table S1. Final Coordinates and Single Point Energy of 4^- After Geometry Optimisation

1.C	-3.366728	1.245940	-6.790820
2.C	-3.197211	-1.197254	-6.202086
3.C	-2.485759	0.167556	-6.124699
4.C	0.087048	2.366680	-6.054240
5.C	0.524618	-1.182617	-5.153799
6.C	3.835118	2.121723	-4.965531
7.C	-0.652236	2.256744	-4.705217
8.C	-1.454109	3.549588	-4.456300
9.C	4.209798	-0.182107	-4.021766
10.C	-0.403674	-0.784386	-3.988967
11.C	3.535985	1.182445	-3.778755
12.C	-3.944038	1.596816	-3.290808
13.C	-5.183531	0.710792	-3.188680
14.C	-1.059754	-2.046475	-3.404761
15.C	6.684138	2.507616	-2.671730
16.C	2.074181	4.203424	-2.502619
17.C	5.364667	3.133529	-2.170940
18.C	-2.746659	-4.972131	-1.953402
19.C	-5.757597	-1.234185	-1.785354
20.C	-4.558131	-2.173377	-1.869788
21.C	2.410700	-2.803454	-1.775165
22.C	1.552468	-5.149514	-1.382814
23.C	2.282111	2.990973	-1.573704
24.C	5.651455	4.043685	-0.961213
25.C	-6.055061	1.044383	-0.905913
26.C	-5.144741	4.890027	-0.742256
27.C	5.220743	-0.110272	-0.834845
28.C	5.391523	-4.629266	-0.852843
29.C	1.877286	-3.794553	-0.724646
30.C	-1.762209	3.819813	-0.729208
31.C	-2.482879	-4.749730	-0.450437
32.C	-6.074133	-4.893310	-0.132466
33.C	2.208654	3.446824	-0.107739
34.C	-2.484341	-6.113559	0.269375
35.C	4.547500	-5.058701	0.361644
36.C	6.057467	0.202941	0.404812
37.C	-5.407839	1.029653	0.477337
38.C	-5.221354	4.360425	0.702987
39.C	-2.038866	3.712886	0.780881
40.C	-5.240606	-4.152080	0.933354
41.C	-5.409904	5.549663	1.670498
42.C	5.479869	-5.433188	1.534000
43.C	-1.635305	5.019097	1.489893
44.C	-6.155933	-3.230472	1.762400
45.C	5.595997	-1.599784	2.029273
46.C	2.089963	-6.459731	2.150001
47.C	-2.643041	-2.790867	1.979243
48.C	2.145498	-4.922475	2.255586
49.C	0.740650	-4.366252	2.530449

50.C	4.299028	-2.253254	2.504435
51.C	4.342602	1.807385	2.633878
52.C	-3.042579	-1.401729	2.501411
53.C	5.445258	0.756303	2.731198
54.C	-2.736137	-3.802777	3.139436
55.C	-3.948159	2.993258	3.138295
56.C	2.381197	4.498348	3.355353
57.C	-5.301823	2.466893	3.657949
58.C	-2.797025	2.201699	3.775739
59.C	1.377407	3.458153	3.889855
60.C	0.850866	-0.957124	3.948968
61.C	0.582107	0.493608	4.377021
62.C	0.606760	4.052803	5.085466
63.C	3.057329	1.649194	5.860413
64.C	-0.040165	0.504215	5.788773
65.C	4.109893	2.745520	6.126808
66.C	3.628047	0.271690	6.251849
67.H	-3.565812	0.984465	-7.846400
68.H	-3.566022	-1.388375	-7.226855
69.H	-2.899407	2.241369	-6.783383
70.H	-0.618265	2.445053	-6.897746
71.H	-1.577435	0.083849	-6.755963
72.H	-4.347472	1.336644	-6.297761
73.H	0.743935	1.509392	-6.254562
74.H	0.719224	3.272026	-6.078664
75.H	3.499795	1.668539	-5.914812
76.H	-0.038014	-1.604653	-6.002673
77.H	-2.533869	-2.030234	-5.934654
78.H	-4.070431	-1.244532	-5.531654
79.H	1.111875	-0.335986	-5.533325
80.H	4.915602	2.314882	-5.066320
81.H	-2.224339	3.710563	-5.227977
82.H	3.876352	-0.614766	-4.981245
83.H	3.335970	3.096880	-4.878406
84.H	1.243286	-1.952785	-4.827702
85.H	-0.788004	4.429167	-4.480441
86.H	-3.939062	2.080345	-4.281931
87.H	5.307321	-0.094267	-4.075129
88.H	-5.063363	-0.129356	-3.886106
89.H	0.115395	2.207067	-3.909302
90.H	-1.774192	-2.507703	-4.101269
91.H	2.444907	0.994801	-3.773761
92.H	6.541396	1.856745	-3.546287
93.H	-6.108779	1.254905	-3.469803
94.H	-1.954401	3.540860	-3.481053
95.H	1.980848	3.912437	-3.557961
96.H	7.401798	3.297008	-2.962410
97.H	3.968197	-0.902396	-3.231232
98.H	0.226972	-0.358751	-3.183204
99.H	5.010691	3.796727	-2.986222

100.H	-0.299998	-2.807566	-3.167160
101.H	-6.491008	-1.422970	-2.596158
102.H	-4.032467	-1.979403	-2.822906
103.H	-4.049488	2.425134	-2.565827
104.H	1.758705	-2.787153	-2.661923
105.H	0.861041	-5.012610	-2.231984
106.H	-1.999119	-5.666094	-2.375905
107.H	-2.688919	-4.039165	-2.524641
108.H	2.903347	4.926437	-2.427410
109.H	-1.597916	-1.862352	-2.460740
110.H	1.150489	4.742488	-2.234327
111.H	-3.736747	-5.419589	-2.137042
112.H	7.174776	1.907733	-1.888902
113.H	-4.929792	-3.208107	-1.948000
114.H	3.423792	-3.053389	-2.118788
115.H	5.827492	0.094567	-1.731669
116.H	2.456295	-5.642839	-1.777050
117.H	4.794270	-4.564022	-1.772389
118.H	1.431587	2.296340	-1.725947
119.H	-4.979571	4.091824	-1.479553
120.H	6.508717	4.710649	-1.166682
121.H	-6.013434	2.071600	-1.293667
122.H	-6.076643	5.415119	-1.020399
123.H	2.430917	-1.762707	-1.410107
124.H	1.073456	-5.848848	-0.681752
125.H	-2.381242	4.591449	-1.209350
126.H	-6.265781	-1.408637	-0.827078
127.H	6.203051	-5.356190	-1.042003
128.H	-7.121601	0.740222	-0.870520
129.H	-4.323068	5.614311	-0.856602
130.H	-1.942798	2.876759	-1.269147
131.H	-6.567254	-4.194978	-0.827018
132.H	4.794840	4.683554	-0.709426
133.H	5.028215	-1.199359	-0.855895
134.H	-5.470628	-5.587986	-0.734251
135.H	-0.708186	4.077735	-0.916377
136.H	5.866670	-3.647116	-0.699591
137.H	0.928177	-3.367987	-0.346322
138.H	-1.789625	-6.814051	-0.227555
139.H	-1.459375	-4.334523	-0.372999
140.H	5.902924	3.464448	-0.058001
141.H	4.029210	-5.994476	0.068873
142.H	-3.482435	-6.582113	0.250023
143.H	-6.876736	-5.484399	0.345526
144.H	3.041292	4.109711	0.165196
145.H	7.053107	-0.284664	0.368956
146.H	1.275895	4.000940	0.082173
147.H	6.218255	1.289058	0.446581
148.H	2.210012	2.612023	0.611571
149.H	-6.146359	3.754338	0.771662

150.H	-5.420396	-0.006053	0.862945
151.H	-6.258032	6.182030	1.350502
152.H	-6.054716	1.603547	1.163341
153.H	-2.173130	-6.042962	1.320530
154.H	5.960411	-2.155517	1.154454
155.H	6.149559	-6.264493	1.247625
156.H	-2.224209	5.876773	1.126818
157.H	-6.524031	-2.378642	1.168515
158.H	-0.574615	5.253350	1.302379
159.H	1.487558	-6.785574	1.287417
160.H	-1.380107	2.905874	1.161347
161.H	-4.518504	6.197013	1.698156
162.H	-4.865909	-4.932134	1.627053
163.H	4.455378	2.339094	1.671129
164.H	3.083976	-6.919904	2.051360
165.H	-1.580680	-2.717140	1.680472
166.H	0.039393	-4.661504	1.734716
167.H	-7.043240	-3.780056	2.126269
168.H	6.127183	-4.590852	1.824337
169.H	-2.812531	-0.586871	1.794910
170.H	4.930590	-5.751554	2.431926
171.H	0.736157	-3.270294	2.578620
172.H	-5.613296	5.227405	2.701868
173.H	2.914931	4.139582	2.467709
174.H	-1.769276	4.961795	2.580963
175.H	6.389958	-1.628247	2.804256
176.H	-5.644636	-2.816269	2.642205
177.H	6.455315	1.215379	2.739592
178.H	1.615516	-6.891574	3.049815
179.H	4.536355	-3.246352	2.926451
180.H	-4.111612	-1.328908	2.747677
181.H	-2.404190	-4.809863	2.855970
182.H	-6.161581	3.018669	3.248150
183.H	2.768955	-4.681737	3.139283
184.H	0.977850	-1.044402	2.855315
185.H	-3.766212	-3.890140	3.521316
186.H	3.903479	-1.663985	3.352068
187.H	4.522662	2.569513	3.410440
188.H	1.857285	5.427799	3.071826
189.H	0.645127	3.283866	3.077721
190.H	-5.438031	1.404026	3.405720
191.H	0.334196	-4.747942	3.482638
192.H	-3.854315	4.042741	3.478174
193.H	-2.475814	-1.160720	3.415627
194.H	5.318417	0.210638	3.676040
195.H	-2.786410	1.159699	3.426456
196.H	-1.816842	2.635944	3.534269
197.H	3.133291	4.772586	4.112055
198.H	-0.177837	0.886429	3.677885
199.H	-2.104588	-3.477209	3.982548

200.H	1.729561	-1.401060	4.438164
201.H	-5.355580	2.544141	4.758305
202.H	-0.014676	-1.598579	4.183218
203.H	-2.889020	2.181120	4.875676
204.H	0.183134	5.038207	4.822509
205.H	5.017396	2.606818	5.517616
206.H	-0.231232	3.418123	5.404088
207.H	3.726204	3.755413	5.923699
208.H	4.469376	-0.017246	5.602349
209.H	-0.977164	-0.077572	5.797922
210.H	1.261916	4.206166	5.959060
211.H	-0.281622	1.515845	6.142777
212.H	2.877745	-0.528651	6.187162
213.H	0.631233	0.048535	6.533973
214.H	2.222375	1.853921	6.560487
215.H	4.432305	2.723919	7.184327
216.H	4.010959	0.283442	7.288929
217.N	-2.721407	0.806025	-3.022594
218.N	-5.288327	0.167755	-1.816762
219.N	3.950020	0.651983	-0.804985
220.N	-3.656880	-1.965342	-0.710801
221.N	-4.024627	1.553020	0.406313
222.N	3.322817	-2.310873	1.392876
223.N	5.314033	-0.207021	1.616792
224.N	3.006311	1.172187	2.740483
225.P	-0.036534	-0.005506	0.078466
226.Si	-1.653689	0.617027	-4.413760
227.Si	3.830000	1.934844	-2.008795
228.Si	-3.551355	-3.349539	0.374715
229.Si	3.007947	-3.945232	0.818781
230.Si	-3.807115	3.106030	1.210134
231.Si	2.092480	1.674452	4.161344
232.U	-2.640960	0.118585	-0.804268
233.U	2.580854	-0.159534	0.877401

Energy: – 1255.42505844 eV

Table S2. Single Point Energy and Coordinates used for 7

C	-4.52929	2.27629	-5.61663
C	-2.91331	-1.05550	-5.20203
C	-2.13448	0.18444	-4.75387
C	3.64474	-2.93299	-4.56024
C	-4.67008	1.73175	-4.18828
C	-0.71066	-0.20856	-4.33797
C	-1.46625	3.41130	-3.99006
C	2.84988	-5.04468	-3.46653
C	-5.46940	2.73755	-3.34861
C	2.85100	-3.51816	-3.37751
C	1.40733	-2.99963	-3.44559

C	4.60617	3.75091	-3.11161
C	2.03676	2.17438	-2.97011
C	-1.92135	2.54249	-2.82432
C	2.02057	5.77917	-2.45874
C	5.11462	-0.68480	-2.43329
C	-4.55965	-0.76182	-2.10307
C	-1.83886	-3.26602	-1.92405
C	5.33798	-3.91441	-1.66122
C	-2.46848	3.41746	-1.71785
C	6.26386	-0.53973	-1.46691
C	2.33777	4.78865	-1.34201
C	-5.63108	-0.30135	-1.14734
C	-0.76537	-5.34693	-1.02883
C	6.18134	1.76219	-0.63888
C	0.98939	4.33924	-0.68148
C	-1.18570	-3.91452	-0.70165
C	-4.37412	-5.23336	-0.36426
C	3.12853	5.51759	-0.22841
C	2.68264	-3.39550	-0.31375
C	5.06422	2.60669	-0.05450
C	-5.40308	-1.72808	0.83866
C	-3.56265	-5.12864	0.92666
C	6.37307	-0.11638	0.94295
C	-4.60940	1.88182	0.98503
C	-5.57409	0.72163	1.10625
C	-3.06397	-6.53239	1.36126
C	-2.52447	4.91819	1.48872
C	5.36997	-1.07307	1.58256
C	-4.22940	-2.08477	1.72783
C	-1.56640	3.83066	2.01833
C	0.08552	-4.75860	2.38336
C	-1.05919	-3.87265	2.36971
C	-0.45043	4.47183	2.88378
C	2.42876	1.59229	2.96217
C	-1.86742	-3.89569	3.68436
C	-1.31755	1.21652	3.64678
C	-3.87124	3.00667	3.92041
C	5.21728	1.11472	4.03052
C	-1.79990	-0.22218	3.79932
C	1.80338	-1.63193	3.96712
C	3.10507	-1.00278	4.49966
C	-4.64299	1.89290	4.64310
C	4.13911	-2.11207	4.72674
C	-3.41117	4.05587	4.95325
C	-0.86221	1.73033	5.01313
C	2.77939	-0.36001	5.87519
H	-4.05349	1.62220	-6.17003
H	-5.41927	2.43743	-5.99396
H	-2.42089	-1.50976	-5.91724
H	-4.02542	3.11649	-5.59786

H	-3.79526	-0.78519	-5.53354
H	-2.06265	0.79840	-5.53987
H	3.35109	-3.35800	-5.39302
H	-0.30377	-0.75522	-5.04222
H	-1.12943	2.83994	-4.71150
H	3.48747	-1.96702	-4.61332
H	4.60099	-3.10139	-4.42702
H	-3.02239	-1.66493	-4.44250
H	-5.25301	0.92304	-4.26531
H	-2.22321	3.94003	-4.31845
H	2.23857	-5.33007	-4.17726
H	0.96482	-3.36835	-4.23843
H	-0.17496	0.60070	-4.20207
H	4.23590	4.28134	-3.84769
H	-6.39625	2.76439	-3.66581
H	3.75535	-5.36076	-3.66810
H	-0.75299	4.01284	-3.69008
H	-5.06842	3.62741	-3.43564
H	5.04832	2.95455	-3.47303
H	1.58961	2.73671	-3.63647
H	1.41412	-2.02115	-3.50069
H	-0.74412	-0.72217	-3.50402
H	2.54038	1.46719	-3.42461
H	5.37618	-1.28603	-3.17507
H	1.46827	5.33937	-3.13851
H	2.85578	6.08966	-2.86708
H	-4.90912	-0.74501	-3.02933
H	4.88825	0.19927	-2.81752
H	5.25759	4.28966	-2.61606
H	2.55536	-5.42159	-2.61127
H	-1.31371	-3.47515	-2.72459
H	5.93328	-3.66051	-2.39711
H	0.92201	-3.27825	-2.64115
H	-5.45392	2.46284	-2.40795
H	-1.09502	2.11541	-2.45739
H	1.53404	6.54487	-2.08789
H	1.36616	1.77051	-2.38047
H	7.04221	-0.13364	-1.92453
H	-3.26740	3.88675	-2.03727
H	-2.74914	-3.61311	-2.03081
H	5.14906	-4.87470	-1.71029
H	-4.29809	-1.69210	-1.88830
H	-0.17391	-5.34340	-1.81025
H	6.25300	1.91220	-1.61474
H	-1.87224	-2.29451	-1.79983
H	-5.89288	0.63010	-1.35744
H	-1.78919	4.07225	-1.45322
H	-6.43169	-0.87713	-1.23495
H	-1.56125	-5.88431	-1.22381
H	6.53233	-1.42876	-1.12413

H	0.44496	3.86242	-1.34227
H	-3.81086	-5.60489	-1.07493
H	5.77020	-3.70981	-0.80588
H	-2.70337	2.86022	-0.94664
H	3.97131	5.85773	-0.59524
H	-4.68639	-4.34253	-0.62709
H	-5.14556	-5.82005	-0.21842
H	7.04684	1.99609	-0.21861
H	-0.34216	-3.40566	-0.52985
H	2.38419	-4.32540	-0.39504
H	0.50208	5.12821	-0.36481
H	5.24893	3.56450	-0.22357
H	-0.29122	-5.73137	-0.26209
H	1.90211	-2.80492	-0.26522
H	2.59582	6.26556	0.11411
H	-5.51588	-2.41020	0.13009
H	-4.57148	2.18545	0.04345
H	1.17830	3.74615	0.07538
H	3.31870	4.89094	0.50068
H	-2.37725	-6.84341	0.73510
H	3.21953	-3.29481	0.50004
H	7.24116	-0.57338	0.80987
H	-6.48293	1.00817	0.83783
H	5.02176	2.46947	0.92496
H	-3.20853	4.50339	0.92237
H	-2.01796	5.57205	0.96335
H	5.27026	-1.87840	1.01545
H	-3.81597	-7.16110	1.36016
H	-6.23641	-1.68691	1.37132
H	-1.10062	3.47193	1.20934
H	6.51889	0.66626	1.53139
H	-4.92710	2.63760	1.53986
H	-4.21353	-4.84477	1.63087
H	0.66431	-4.55632	1.61882
H	-5.61014	0.40881	2.04478
H	-2.68394	-6.47946	2.26292
H	-2.95569	5.36957	2.24410
H	-4.41102	-2.94056	2.19122
H	-0.21994	-5.68799	2.32506
H	0.00397	5.16919	2.36636
H	5.70312	-1.35977	2.46969
H	-4.10645	-1.38223	2.41442
H	1.74868	1.24951	2.34550
H	-0.63638	-2.96652	2.38705
H	2.85571	2.38286	2.57105
H	-1.83451	-0.64999	2.91834
H	0.19703	3.78377	3.14409
H	0.58736	-4.63128	3.21541
H	-4.54094	3.47044	3.34035
H	-0.49586	1.17448	3.07865

H	1.99719	-2.14537	3.15532
H	5.49200	1.86819	3.46738
H	-0.84735	4.86774	3.68753
H	-2.38685	-4.72542	3.73414
H	-2.47587	-3.12785	3.70745
H	4.34066	-2.55013	3.87361
H	1.15915	-0.92327	3.75948
H	2.00584	1.83616	3.81202
H	-4.92701	1.21724	3.99254
H	5.95415	0.47215	4.09856
H	-2.69427	-0.22618	4.19985
H	-2.98957	4.80882	4.48843
H	-1.25312	-3.84876	4.44643
H	-1.18087	-0.71463	4.37758
H	1.42601	-2.22718	4.64782
H	4.98601	1.44246	4.92467
H	-5.43135	2.27423	5.08315
H	4.95918	-1.72347	5.09618
H	-0.61807	2.67682	4.94125
H	-4.18590	4.37724	5.45991
H	-4.06329	1.47644	5.31470
H	3.77794	-2.77155	5.35513
H	-0.08501	1.21440	5.31381
H	-2.76542	3.64834	5.56754
H	-1.59142	1.62926	5.65991
H	2.11927	0.35410	5.75407
H	3.59798	0.01418	6.26282
H	2.41564	-1.04268	6.47692
N	-3.37740	0.12700	-1.99842
N	3.94635	-1.23571	-1.73885
N	3.76317	2.23425	-0.66714
N	5.82923	0.32048	-0.35185
N	-5.10733	-0.37721	0.22317
N	-2.99795	-2.20962	0.91845
N	-3.25647	1.47107	1.43050
N	4.06182	-0.40915	1.73506
P	0.29219	0.01106	-0.06787
Si	-3.04988	1.12151	-3.36909
Si	3.21318	3.22519	-1.95263
Si	3.71317	-2.94209	-1.79749
Si	-2.23474	-3.75821	0.87235
Si	-2.52290	2.35865	2.74151
Si	3.71879	0.27241	3.26433
U	2.99961	0.13504	-0.18810
U	-2.35559	-0.16100	0.06886

Energy: – 1074.35800173 eV

References

1. B. M. Gardner, P. A. Cleaves, C. E. Kefalidis, J. Fang, L. Maron, W. Lewis, A. J. Blake, S. T. Liddle, *Chem. Sci.* **2014**, *5*, 2489.
2. R. Boaretto, P. Roussel, N. W. Alcock, A. J. Kingsley, I. J. Munslow, C. J. Sanders, P. Scott, *J. Organomet. Chem.* **1999**, *591*, 174.
3. R. Boaretto, P. Roussel, A. J. Kingsley, I. J. Munslow, C. J. Sanders, N. W. Alcock, P. Scott, *Chem. Commun.* **1999**, 1701.
4. B. M. Gardner, G. Balázs, M. Scheer, F. Tuna, E. J. L. McInnes, J. McMaster, W. Lewis, A. J. Blake, S. T. Liddle, *Angew. Chem. Int. Ed*. **2014**, *53*, 4484.
5. R. Klement, G. Brauer, *Handbuch der Präparativen Anorganischen Chemie*, 3rd Ed, Ferdinand Enke Verlag, Stuttgart, **1975**, Issue 1, pp 516.
6. P. J. Bailey, R. A. Coxall, C. M. Dick, S. Fabre, L. C. Henderson, C. Herber, S. T. Liddle, D. Loroño-González, A. Parkin, S. Parsons, *Chem. Eur. J.* **2003**, *9*, 4820.
7. P. B. Hitchcock, M. F. Lappert, L. Maron, A. V. Protchenko, *Angew. Chem. Int. Ed.* **2008**, *47*, 1488.
8. C. Fonseca Guerra, J. G. Snijders, G. te Velde E. J. Baerends, *Theor. Chem. Acc.* **1998**, *99*, 391.
9. G. te Velde, F. M. Bickelhaupt, S. J. A. van Gisbergen, C. Fonseca Guerra, E. J. Baerends, J. G. Snijders T. Ziegler, *J. Comput. Chem.* **2001**, *22*, 931.
10. S. H. Vosko, L. Wilk, M. Nusair, *Can. J. Phys.* **1980**, *58*, 1200.
11. A. D. Becke, *Phys. Rev. A.* **1988**, *38*, 3098.
12. J. P. Perdew, *Phys. Rev. B.* **1986**, *33*, 8822.
13. S. Portmann, H. P. Luthi, *Chimia* **2000**, *54*, 766.
14. NBO 5.0: E. D. Glendening, J. K. Badenhoop, A. E. Reed, J. E. Carpenter, J. A. Bohmann, C. M. Morales, F. Weinhold, (Theoretical Chemistry Institute, University of Wisconsin, Madison, WI, 2001); http://www.chem.wisc.edu/~nbo5.
15. R. F. W. Bader, *Atoms in Molecules: A Quantum Theory*, Oxford University Press, New York, 1990.
16. R. F. W. Bader, *J. Phys. Chem. A.* **1998**, *102*, 7314.
17. http://www.quimica.urv.es/XAIM.
